# Closing the Loop After Positive Fecal Immunochemical Test (FIT) Results: Barriers to Colonoscopy Completion Within the Saudi Model of Care in Qassim Health Cluster

**DOI:** 10.7759/cureus.107266

**Published:** 2026-04-17

**Authors:** Fatmah Alribdi, Mohammed Awad Alanazi, Ahmed A Almeman, Abdulrahman Mesned, Abdulsalam A Alfawzan, Annalyn V Camba, Khuzama Alkhalaf, Albandary Alanazi, Ruba S Alsheban, Aseel A Almesned, Fatimah S Alriyaee, Abdullah Al Qwaee

**Affiliations:** 1 Saudi Model of Care, Qassim Health Cluster, Buraidah, SAU; 2 Department of Pharmacology, College of Medicine, Qassim University, Buraidah, SAU; 3 Department of Pediatric Cardiology, Prince Sultan Cardiac Center, Riyadh, SAU; 4 College of Medicine, Sulaiman Al Rajhi University, Al Bukayriyah, SAU; 5 College of Medicine, Qassim University, Buraidah, SAU

**Keywords:** colorectal cancer (crc) screening, fecal immunochemical test (fit), health care services, patient barriers, qassim region, saudi arabia

## Abstract

Background and aim

In the Qassim Region of Saudi Arabia, a significant proportion of patients with a positive fecal immunochemical test (FIT) do not proceed to diagnostic colonoscopy, compromising colorectal cancer (CRC) screening efforts. The specific reasons behind this drop-off remain poorly characterized. This study aimed to identify and evaluate the multidimensional barriers, including knowledge gaps, psychosocial factors, and sociodemographic traits, that prevent patients from undergoing follow-up colonoscopy after a positive FIT.

Methods

A cross-sectional study was conducted between January and October 2024 among 212 nonadherent patients in the Qassim Region. Data were collected using a structured questionnaire. Descriptive statistics were utilized to evaluate barriers, and a multivariable logistic regression analysis was performed to identify sociodemographic predictors of psychological barriers.

Results

The cohort was predominantly male (160; 75.47%), with a university-level education (104; 49.06%). A significant knowledge gap regarding colonoscopy was identified. The most frequently reported barriers were embarrassment (65; 30.95%), fear of a cancer diagnosis (43; 20.48%), and the misconception of not needing the test due to being asymptomatic (32; 15.24%). Multivariable regression indicated that higher educational attainment showed a trend toward a lower likelihood of reporting psychological barriers (OR: 0.56, p = 0.078). Additionally, gender was significantly associated with reporting family history (p = 0.004) and perceiving screening as important (p = 0.048).

Conclusions

Nonadherence to follow-up colonoscopy after a positive FIT in the Qassim region is primarily driven by psychosocial stigma (embarrassment and fear) and procedural knowledge deficits. These barriers can be minimized through targeted continuous patient education and culturally sensitive psychological support, which are essential to bridging gaps in the CRC screening cascade.

## Introduction

Colorectal cancer (CRC) represents a very different picture among neoplastic diseases, as it can be largely prevented by timely screening. Even so, it continues to contribute a substantial burden of illness and death across the world. CRC is the world’s third most diagnosed cancer and is ranked as the second leading cause of cancer-related death. It is responsible for one in 10 cancer cases and for a similar proportion of cancer deaths [1].

The global distribution of CRC burden shows significant heterogeneity. It is predominantly reported in industrialized countries. Although many developed countries have made substantial progress in reducing the incidence of CRC and related deaths, largely due to advancements in screening and treatment, a concerning trend is the significant increase in CRC among individuals below 50 years of age [2,3]. This change is especially associated with socioeconomic conditions and resource inequalities, leading to late-stage detection, emphasizing the need for comprehensive and effective early detection strategies [4].

The natural history of CRC provides an important opportunity for prevention, as it progresses gradually over approximately 10-15 years from adenomatous growth to invasive malignant disease. This premalignant stage allows for effective clinical intervention [5]. Current CRC screening methodologies are based on a stepwise approach that begins with stool-based testing. The fecal immunochemical test (FIT) successfully detects occult human blood in stool. This test is noninvasive, user-friendly, and relatively low-cost, and due to its high precision and accuracy, it has become a widely accepted tool for screening programs [6].

A positive FIT is an indicator for further clinical investigation and not an absolute diagnosis of cancer. Confirmation is performed through colonoscopy, which is considered the gold standard for CRC diagnosis. It allows direct visualization of the colon and enables not only tissue sampling for histological examination but also the removal of precancerous polyps. Hence, it plays a key role in both diagnosis and treatment [7].

The success of this stepwise screening strategy depends largely on patient adherence, which remains a major barrier in global CRC prevention efforts. Even in the United States, despite substantial efforts, approximately 40% of individuals in the recommended age group remain noncompliant with screening guidelines [8]. This nonadherence disproportionately affects younger adults, racial and ethnic minorities, uninsured individuals, and low-income populations, further widening health disparities and contributing to higher disease burden in these groups [9].

Global research has identified multiple barriers to screening adherence at a multidimensional level. Common patient-level barriers include fear of cancer diagnosis, fear of invasive procedures, loss of dignity, low perceived risk due to absence of symptoms, fatalistic health beliefs, and lack of information [10,11]. At the healthcare provider level, weak or inconsistent recommendations from primary care physicians represent an additional barrier [12]. System-level challenges include treatment costs, insurance gaps, transportation limitations, inflexible scheduling, and prolonged wait times [13].

In the Kingdom of Saudi Arabia, CRC epidemiology has changed over time, as it was previously considered a low-incidence region. The population has experienced a continuous and substantial increase in incidence rates. This trend is associated with lifestyle changes, including dietary shifts, reduced physical activity, and rising obesity rates, all of which are linked to economic development [14]. CRC is now the most common cancer among Saudi men and ranks third among women. Its incidence rate has more than doubled since the 1990s [15,16].

Notably, the age at diagnosis is lower than that observed in Western populations. These diagnoses in individuals under 50 years of age place an additional burden on screening efforts within an already strained healthcare system [17]. Regional variation is also evident in the Qassim Region of Central Saudi Arabia, where the proportion of rectal cancer is higher in males than in females, suggesting regional differences in environmental, behavioral, and genetic factors [15].

The Saudi Ministry of Health has established guidelines to address these challenges. These guidelines recommend annual FIT screening starting at age 45 years or direct colonoscopy as an alternative screening strategy [18]. However, awareness of CRC and the availability of screening remain limited in Saudi Arabia. As reported by Almadi and Alghamdi in a national survey, only 27% of participants were aware of screening guidelines. The study also highlighted a widespread misconception that screening is only necessary in the presence of symptoms [19]. Similar findings were reported in Jeddah, where screening options were known to only one-third of the sampled population [20].

This lack of awareness is rooted in cultural and psychosocial factors. In Saudi Arabia and the broader Middle East region, studies have identified key influences, including fear of a cancer diagnosis and its social consequences; reluctance toward procedures involving the pelvic region, particularly among women; and interpretations of illness within religious and spiritual frameworks [21,22]. A national survey conducted in Jordan reported similar findings, identifying social and cultural stigma, fear of diagnosis, and limited access to healthcare facilities as major barriers [23].

Although multiple studies have focused on awareness and behavioral responses, there remains a need to further investigate failure to complete the screening pathway. Limited information is available regarding patients who have already received a positive FIT result but have not proceeded to colonoscopy. These individuals represent a high-risk group who have initiated screening but disengage at a critical stage of the diagnostic pathway. Despite being identified as high risk, many do not proceed to the next step, leading to a breakdown in the screening cascade.

Therefore, this study was designed to identify the specific barriers to completion of the screening program among the population of the Qassim Region. The focus is on individuals with a positive FIT who did not undergo a colonoscopy. The primary aim is to identify, classify, and analyze these barriers. In addition, the study seeks to examine the relationship between demographic factors and patient behavioral characteristics. This will support efforts to complete the screening cascade and address social and cultural determinants that contribute to regional disparities in CRC outcomes.

Objectives

This study aimed to analyze the multidimensional barriers to follow-up colonoscopy among FIT-positive patients in the Qassim Region. The primary objectives included examining associations between nonadherence and sociodemographic variables (age, gender, and education) and evaluating the impact of health beliefs and cultural attitudes on screening behavior. Additionally, the study sought to identify potential facilitators that may motivate nonadherent individuals to undergo colonoscopy. By understanding these underlying factors, targeted public health interventions can be developed to improve screening compliance and reduce the risk of CRC.

## Materials and methods

Study design

This study was conducted using a cross-sectional design and employed a combination of descriptive and analytical methodologies. This design specifically focused on identifying possible barriers to colonoscopy uptake among patients in the Qassim Region. This approach investigated the relationship between these barriers and patients’ attitudes toward completing colonoscopy after a positive FIT result.

Study duration and settings

Data collection was conducted over a period of 10 months, from January to October 2024, in primary health care centers and hospitals in the Qassim Region that participated in the national colon cancer screening program.

Data collection tool

A structured questionnaire was utilized, specifically developed and adapted to reflect the Saudi sociocultural context (Appendix A). The questionnaire underwent a rigorous forward and backward translation process to ensure linguistic and conceptual validity. The instrument comprised five thematic sections: sociodemographics, family oncological history, clinical knowledge of colonoscopy, and a combination of Likert scales and open-ended questions to assess health perceptions. The final section identified potential facilitators and future screening intentions among the target cohort.

Study population and sample size

The study was conducted among adult residents of the Qassim Region who had a positive FIT result between January and October 2024 but remained nonadherent to follow-up colonoscopy. According to records from the Saudi National Colorectal Cancer Screening Program (within the Saudi Model of Care framework), a total of 380 individuals met these criteria during the study period. While the recruitment target was set to encompass this entire eligible population, the final sample consisted of 212 participants who completed the questionnaire, representing a response rate of 55.8%. Given the inherent challenges in engaging a nonadherent population, this sample is considered highly representative of the target group within the regional screening registry.

Inclusion and exclusion criteria

The study included adult residents of the Qassim Region who had a positive FIT result from January to October 2024, had not undergone a colonoscopy, and provided informed consent. The study excluded individuals who had completed a colonoscopy following a positive FIT result to maintain focus on nonadherence. Additionally, those lacking the capacity for informed consent or with inaccurate contact information were excluded, as the latter precluded reliable data collection via telephonic or electronic interviews.

Procedure for data collection

Data were collected through an online laboratory database at participating centers. Online and telephonic interviews were conducted according to the questionnaire, depending on participant preference, after informed consent.

Data analysis

Data were analyzed using IBM SPSS Statistics for Windows, version 28.0 (released 2021; IBM Corp., Armonk, NY, USA). Descriptive statistics were employed to summarize demographic characteristics and questionnaire responses, with categorical variables expressed as absolute numbers and percentages. To evaluate independent predictors of psychological barriers, specifically embarrassment and fear, a multivariable logistic regression model was constructed. This model incorporated sociodemographic variables, including age, gender, educational level, and family history of CRC. Qualitative insights from open-ended questions were categorized using inductive thematic analysis. Statistical significance for all inferential tests was predefined at a p-value of < 0.05.

Ethical considerations

Ethical approval was obtained from the Regional Research Ethics Committee, Qassim Province (approval 607-46-4840). All procedures were based on the Declaration of Helsinki. All verbal and digital informed consents were obtained from participants. Data were stored on password-protected systems, which were accessible only to the research team.

## Results

Participant sociodemographic profile

A sample of 212 patients successfully completed the study (Table 1), with a major proportion being male (160; 75.47%), while the remaining were female (52; 24.53%). Forty-seven participants were below the age of 40, contributing 22.17% of the sample; 51 participants were aged 40-49 years (24.06%); 72 (33.96%) were between 50 and 59 years; and 42 participants were aged 60 years or older (19.81%), respectively. Most participants were highly educated, with 104 (49.06%) holding a university degree. A total of 174 participants (82.08%) were married.

**Table 1:** Sociodemographic profile

Variables	Description	Number (n = 212)	Percentage (%)
Gender	Male	160	75.47
	Female	52	24.53
Age group	Below 40 years	47	22.17
	40-49 years	51	24.06
	50-59 years	72	33.96
	60 years and above	42	19.81
Education	University	104	49.06
	Secondary	60	28.30
	Primary/illiterate	37	17.46
	Intermediate	11	5.19
Marital status	Married	174	82.08
	Single/divorced	38	17.92

Knowledge and awareness assessment

A total of 192 (90.6%) participants were aware of the lifesaving impact of early CRC diagnosis, and 173 (81.6%) considered regular screening to be important or very important. However, knowledge regarding the screening process was notably low, with 154 (72.6%) participants reporting insufficient or no information about colonoscopy. Moreover, 144 (67.9%) participants reported a lack of general awareness regarding CRC screening facilities in the region. Additionally, 135 (63.7%) participants identified physicians as the most trustworthy source of health information.

Prevalence and ranking of perceived barriers

The most frequently reported barrier was embarrassment or shame related to the colonoscopy procedure, identified by 65 (30.95%) participants. This was followed by anxiety regarding a potential cancer diagnosis in 43 (20.48%) individuals and fear of hospital-based procedures in 25 (11.90%). Additionally, 32 (15.24%) participants expressed a lack of perceived need for the procedure due to being asymptomatic. Other identified attitudes included a “live for today” mindset, reported by 10 (4.76%) participants, while four (1.90%) perceived their personal risk of developing CRC as low (Table 2).

**Table 2:** Perceived barriers to colonoscopy uptake ranked by frequency

Rank	Barrier category	Specific reason	Number (n = 212)	Percentage (%)
1	Psychological	Embarrassment or shame about the procedure	65	30.95
2	Anxiety and fear	Fear of receiving a cancer diagnosis (“bad news”)	43	20.48
3	Low perceived need	“I have no symptoms.”	32	15.24
4	Logistical	Busy schedule with no spare time	25	11.90
5	Anxiety and fear	Fear of hospitals or procedures	25	11.90
6	Avoidance	“I prefer to live for today and not worry about the future.”	10	4.76
7	Family history	“No one in my close family has this.”	5	2.38
8	Low perceived risk	“My risk is low.”	4	1.90

Factors that could help patients reconsider

Regarding potential factors for reconsidering colonoscopy, 34 (16.0%) participants indicated they would reconsider if they experienced visible rectal bleeding or a subsequent positive FIT result. Additionally, 33 (15.6%) participants expressed willingness to undergo the procedure if a less invasive diagnostic modality were available. Other facilitating factors included the availability of appointments outside standard working hours for 15 (7.1%) individuals, enhanced public awareness campaigns for 13 (6.1%), and the provision of female endoscopic staff for gender-sensitive cases for eight (3.8%).

Analysis of association with demographic variables

Chi-square tests were applied to examine the associations between gender and other variables. A significant association was found between gender and family history of CRC (Table 3; χ² = 10.883, df = 2, p = 0.004). Similarly, a significant association was observed between gender and the perceived importance of screening (Table 4; χ² = 7.894, df = 3, p = 0.048).

**Table 3:** Association of family history and gender for CRC CRC, colorectal cancer

Family history	Male (n, %)	Female (n, %)	Total (n)
Yes	34 (21.23%)	7 (13.5%)	41
No	99 (61.9%)	44 (84.6%)	143
Don’t know	27 (16.9%)	1 (1.9%)	28

**Table 4:** Relationship between gender and perceived screening importance

Importance	Male (n, %)	Female (n, %)	Total (n)
Very important	95 (59.4%)	28 (53.8%)	123
Important	37 (23.1%)	13 (25%)	50
Not important	24 (15%)	5 (9.6%)	29
Don’t know	4 (2.5%)	6 (11.5%)	10

Predictors of psychological barriers

To evaluate the independent predictors of psychological barriers (such as embarrassment and fear), a multivariable logistic regression model was conducted (Table 5). The analysis revealed that while age, gender, and family history were not statistically significant predictors, a higher educational level (university degree or above) showed a notable trend toward a lower likelihood of reporting psychological barriers (OR: 0.56, 95% CI: 0.30-1.06, p = 0.078). This suggests that education may play a role in mitigating emotional barriers to colonoscopy.

**Table 5:** Multivariable logistic regression analysis of predictors for psychological barriers ^*^ Indicates a statistical trend (0.05 < p < 0.10), highlighting a marginal association between educational level and psychological barriers. CRC, colorectal cancer

Variable	OR	95% CI	p-Value
Gender (female vs. male)	0.86	0.42-1.74	0.677
Age (>50 vs. <50 years)	0.68	0.37-1.25	0.226
Education (university vs. below)	0.56	0.30-1.06	0.078^*^
Family history of CRC (yes vs. no)	0.96	0.46-2.00	0.917

## Discussion

This cross-sectional study elaborates on the factors by which CRC screening is compromised in the Qassim Region of the Kingdom of Saudi Arabia. This study reveals preventable barriers that hinder CRC screening by focusing on patients who stopped undergoing colonoscopy after a positive FIT result. These results paint a broad picture of the region’s population, which endorses the importance of screening for CRC; however, despite this, they are compromised by psychosocial behaviors and fail to convert their knowledge into practice.

The primary hindrance identified was embarrassment and modesty (30.95%), which is strongly associated with regional sociocultural trends. However, this is also a major barrier in screening programs globally [24]. Its predominance in this region indicates a preference for a system based on privacy, bodily respect, and modesty, which are highly valued in pelvic and rectal examinations [25]. This is not merely a preference but a deeply rooted fear that can affect rational behavior and is closely linked to the fear of a cancer diagnosis (20.48%). This represents cultural and social concerns in the population, associated with personal and social implications in the case of positive results [26]. Such diagnoses can be correlated with familial characteristics and social standing in such sensitive societies, which deepens the fear related to diagnosis [27]. These situations require interventions that address not only clinical awareness but also emotional and behavioral factors.

These results show a wide gap between acceptable screening perception and actual implementation. A high proportion (81.6%) placed a high value on screening, and an even larger proportion (90.6%) endorsed the principle of early CRC diagnosis. However, 72.6% reported insufficient knowledge about colonoscopy at the same time. These results also highlight a misconception among 15.24% of participants who firmly believed that “no symptoms mean no need for screening.” This indicates that public health awareness has failed to encourage individuals with positive FIT results to proceed to the colonoscopy cascade. This finding is consistent with previous Saudi research, which indicates that screening awareness remains lower than general disease awareness [19,20].

Gender-based analyses provide further insight for communication strategies. A trend of definitive denial regarding family history of CRC among female participants, alongside emphasis on screening for familial health, may indicate gender-influenced approaches [28]. The gap between basic awareness and its implications across genders suggests that embarrassment, fear, and misinformation are shared challenges. These identified gaps require immediate, tailored interventions.

Interpretation of results on general perception and health beliefs indicates a substantial failure in follow-up screening after a positive FIT. Although participants recognized the benefits of early CRC diagnosis, widespread perceptions still contributed to failure at a critical stage of the screening process. Moreover, the belief that “no symptoms” negate the need for screening underscores an unaddressed gap in procedural understanding [29]. This study emphasizes the need for multifaceted interventions, including reducing barriers, promoting critical health awareness, and improving well-being. Addressing these misconceptions necessitates targeted health education using Information, Education, and Communication (IEC) strategies and focus group discussions (FGDs).

Participants’ suggestions provide future directions to improve reconsideration and enhance screening uptake. Alternative diagnostic techniques, such as CT colonography, indicate a more patient-friendly screening portfolio. These interventions may improve patient comfort and acceptability [30]. Off-hour appointments can play a vital role for individuals with busy schedules who cannot easily allocate time. Patients should be encouraged to treat colonoscopy as an urgent follow-up after recurrent bleeding rather than delaying it as part of routine monitoring.

Limitations

Only patients who consented to participate were included in our study. It is possible that patients who declined to be interviewed had more severe or distinct obstacles, such as extreme social anxiety or complete mistrust of the health care system. As a result, our findings might understate the importance or frequency of some barriers. Highly educated males were dominant in our sample and were more approachable and willing to speak. This may bias the demographic representation of all nonadherent patients in the Qassim region, particularly women and the less educated population. As all data on knowledge, attitudes, and barriers are self-reported, there is a possibility of overreporting socially acceptable barriers, such as being busy, and underreporting less acceptable ones, such as fear and anxiety.

Despite its structure, the instrument might not have fully reflected the complexity and depth of barriers. Some intricate cultural or emotional factors may have been simplified into checkbox responses. Although the open-ended responses were helpful, this investigation would have benefited more from a qualitative interview methodology.

This study was conducted in the Qassim area only and does not depict the complete picture of all Saudi Arabian regions, which may differ greatly in terms of culture, economy, and health care systems. As a result, barrier prevalence and profiles may differ substantially in other regions, limiting generalizability. Our focus was on groups who did not undergo colonoscopy after a positive FIT and did not include a comparison group of patients who underwent colonoscopy following a positive FIT. By comparing these two groups, we could have identified clearer predictors and protective factors, which may have strengthened the findings.

Future recommendations

A multidimensional intervention policy is recommended based on these findings. The health care system should implement patient navigation programs to guide patients with positive FIT results, minimizing emotional barriers and providing logistical support. Preprocedural counseling sessions should be organized to reduce embarrassment, fear, and modesty-related concerns regarding colonoscopy. Furthermore, we strongly recommend the development and distribution of targeted IEC materials to improve community awareness. Engaging patients and various stakeholders through FGDs is also essential to designing effective, culturally acceptable educational interventions. CT colonography should be offered as an alternative to support patient autonomy, and all clinical decision-making should be integrated among physicians. Patients should be offered the option to reschedule appointments after working hours, and gender-specific concerns should be addressed through a dedicated task force. The key role of physicians in patient education and counseling should never be underestimated in completing the FIT-to-colonoscopy cascade.

## Conclusions

This study highlights key human factors that hinder screening continuation in the Qassim Region. These barriers are not primarily due to a lack of awareness but rather a combination of psychological dilemmas and limited understanding of a specific clinical procedure. This situation emphasizes the need to shift toward active intervention frameworks rather than passive, traditional models of patient care in the region. Future strategies should focus on support systems to overcome fear and embarrassment related to colonoscopy, respect for personal rights, flexibility in clinical appointment scheduling, and clear communication about the purpose and steps of screening. This study may motivate patients with positive FIT results to complete their screening journey. It may also help them recognize the protective value of completing screening and the potential to reduce the risk of CRC by following these recommendations.

## Appendices

Appendix A: Study questionnaire 

**Table 6:** Colon study questionnaire This questionnaire was developed by the authors specifically for this study, incorporating elements gathered through a literature review and clinical brainstorming. Credits: Fatmah Alribdi and Mohammed Awad Alanazi

English	Arabic	No.
General information and demographics	المعلومات العامة	Section 1
Age: - Less than 40 - 40 - 49 - 50 - 59 - 60 - 70 - Older than 70	العمر: - أقل من 40 سنة - 40 - 49 سنة - 50 - 59 سنة - 60 - 70 سنة - أكبر من 70 سنة	1
Gender: - Male - Female	الجنس: - ذكر - أنثى	2
Educational level: - Illiterate - Primary or less - Middle school - High school - University or higher	المستوى التعليمي: - أمي (لا أقرأ ولا أكتب) - ابتدائي أو أقل - متوسط - ثانوي - جامعي أو أعلى	3
Marital status: - Single - Married - Widowed - Divorced/separated	الحالة الاجتماعية: - أعزب - متزوج - أرمل - منفصل	4
Nationality: - Saudi - Non-Saudi	الجنسية: - سعودي - غير سعودي	5
Employment status: - Employed - Unemployed	الحالة الوظيفية: - يعمل - لا يعمل	6
Have any of your relatives been diagnosed with colorectal cancer? - Yes - No - Not sure	هل أصيب أحد من أقاربك بسرطان القولون أو المستقيم؟ - نعم - لا - غير متأكد	7
Do you feel you have sufficient information about colonoscopy? - Yes - No	هل تشعر أن لديك معلومات كافية حول فحص القولون بالمنظار؟ - نعم - لا	8
Knowledge and awareness assessment	قياس المعرفة والوعي	Section 2
Do you think there is a lack of awareness about the importance of colorectal cancer screening? - Yes - No	هل تعتقد أن هناك نقص في التوعية حول أهمية الفحص للكشف عن سرطان القولون؟ - نعم - لا	9
In your opinion, how important is regular screening for colorectal cancer? - Very important - Important - Not important - I don't know	ما مدى أهمية الفحص المنتظم للكشف عن سرطان القولون في رأيك؟ - مهم جداً - مهم - غير مهم - لا أعرف	10
Do you believe that early detection can save someone's life from colorectal cancer? - Yes - No - Not sure	هل تعتقد أن الكشف المبكر يمكن أن ينقذ حياة شخص ما من سرطان القولون؟ - نعم - لا - غير متأكد	11
What source of information do you consider the most reliable regarding colorectal cancer? - Doctors - Internet - Family and friends - Other sources	ما هو مصدر المعلومات الذي تعتقد أنه الأكثر موثوقية حول سرطان القولون؟ - الأطباء - الإنترنت - العائلة والأصدقاء - مصادر أخرى	12
Barriers and facilitators for screening	عوائق ومحفزات الفحص	Section 3
What are the reasons that led you to refuse the colonoscopy screening? (Select all that apply) - Not feeling the need for the screening - Fear of test results - Lack of trust in test accuracy - Previous negative experience with other tests - Fear of pain or discomfort during the test - Lack of information about the test - I don't need it because I don't have any symptoms - I don't need it because I am young - I had a similar test previously - Fear of receiving bad news - Feeling embarrassed about the test - Difficulty accessing the testing location - Religious reasons - Lack of time due to a busy schedule - Fear of hospital facilities - I believe my risk of colorectal cancer is low - No one in my close family has colorectal cancer - I don't like undergoing endoscopic tests - I want to live for today and not worry about the future - Other	ما هي الأسباب التي تدفعك لرفض فحص القولون بالمنظار للكشف عن سرطان القولون؟ - عدم الشعور بالحاجة إلى الفحص - خوف من نتائج الفحص - عدم الثقة في دقة الفحوصات - تجربة سلبية سابقة مع فحوصات أخرى - تخوف من الألم أو عدم الراحة أثناء الفحص - نقص المعلومات حول الفحص - لا أحتاجه لأنني لا أشعر بأي أعراض - لا احتاجه لأنني صغير في السن - أجريت فحصا مشابها سابقا - الخوف من تلقي أخبار سيئة - الشعور بالإحراج بشأن الفحص - صعوبة الوصول إلى موقع الفحص - لأسباب دينية - عدم وجود وقت بسبب جدولي المزدحم - الخوف من مرافق المستشفى - أعتقد أن خطر إصابتي بسرطان القولون والمستقيم صغير - لا يوجد أحد في عائلتي القريبة مصاب بسرطان القولون والمستقيم - لا أحب إجراء اختبار المنظار - أريد أن أعيش اليوم ولا أفكر فيما قد يجلبه المستقبل - أخرى	13
If there are other reasons that led you to refuse, please write them here: - (Open-ended question)	اذا كان هناك أسباب أخرى دفعتك للرفض فضلا كتابتها هنا: - [إجابة مفتوحة]	14
What might make you reconsider your decision regarding undergoing colonoscopy? - Increased awareness of the importance of screening - Better information about the test and its procedures - Information about the importance of early detection - Personal experiences of other people - Psychological support from healthcare providers - Free transportation to the testing facility - Appointments outside of working hours - Sick leave to undergo the test - Counseling session with a healthcare provider - Other	ما الذي يمكن أن يجعلك تعيد النظر في قرارك بشأن فحص القولون بالمنظار للكشف عن سرطان القولون؟ - زيادة التوعية بأهمية الفحص - تحسين المعلومات حول الفحص وإجراءاته - معلومات عن أهمية الفحص للكشف المبكر - تجارب شخصية لأشخاص آخرين - الدعم النفسي من مقدمي الرعاية الصحية - نقل مجاني إلى مكان سحب العينة - موعد خارج ساعات العمل - إجازة مرضية لإجراء التحليل - جلسة استشارية مع مقدم رعاية صحية - أخرى	15
